# The adjunctive use of antimicrobial photodynamic therapy, light-emitting-diode photobiomodulation and ozone therapy in regenerative treatment of stage III/IV grade C periodontitis: a randomized controlled clinical trial

**DOI:** 10.1007/s00784-024-05794-0

**Published:** 2024-07-12

**Authors:** Deniz Ozbay Cetiner, Sila Cagri Isler, Rahsan Ilikci-Sagkan, Janset Sengul, Ozlem Kaymaz, Ahu Uraz Corekci

**Affiliations:** 1 Department of Periodontology, Faculty of Dentistry, Gazi University, Biskek cad. 1. Sokak No:4, Emek Ankara, 06490 Turkey; 2 Department of Periodontology, School of Dental Medicine, University of Bern, Bern, Switzerland; 3 Department of Medical Biology, School of Medicine, Uşak University, Uşak, 64300 Turkey; 4 Private Office, Memorial Hospital, Ankara, Turkey; 5 Department of Statistic, Faculty of Science, Ankara University, Ankara, Turkey; 6 Department of Periodontology, Faculty of Dentistry, Izmir Demokrasi University, Izmir, Turkey

**Keywords:** Grade C periodontitis, Guided tissue regeneration, Photodynamic therapy, Photobiomodulation therapy, Ozone

## Abstract

**Objectives:**

To assess the short-term efficacy of multiple sessions of antimicrobial photodynamic therapy (aPDT), light-emitting-diode (LED) photobiomodulation, and topical ozone therapy applications following surgical regenerative treatments on clinical parameters, patient-centered outcomes, and mRNA expression levels of VEGF, IL-6, RunX2, Nell-1, and osterix in gingival crevicular fluid samples in patients with stage III/IV, grade C periodontitis.

**Materials and methods:**

Forty-eight systemically healthy patients were assigned into four groups to receive adjunctive modalities with regenerative periodontal surgical treatment. A 970 ± 15 nm diode laser plus indocyanine-green for aPDT group, a 626 nm LED for photobiomodulation group, and topical gaseous ozone were applied at 0, 1, 3, and 7 postoperative days and compared to control group. The clinical periodontal parameters, early wound healing index (EHI), and postoperative patients’ morbidity were evaluated. The mRNA levels of biomarkers were assessed by real-time polymerase chain reaction.

**Results:**

No significant difference in the clinical parameters except gingival recession (GR) was identified among the groups. For group-by-time interactions, plaque index (PI) and probing pocket depths (PD) showed significant differences (*p* = 0.034; *p* = 0.022). In sites with initial PD > 7 mm, significant differences were observed between control and photobiomodulation groups in PD (*p* = 0.011), between control and aPDT, and control and photobiomodulation groups in CAL at 6-month follow-up (*p* = 0.007; *p* = 0.022). The relative osterix mRNA levels showed a statistically significant difference among the treatment groups (*p* = 0.014).

**Conclusions:**

The additional applications of aPDT and LED after regenerative treatment of stage III/IV grade C periodontitis exhibited a more pronounced beneficial effect on clinical outcomes in deep periodontal pockets.

**Supplementary Information:**

The online version contains supplementary material available at 10.1007/s00784-024-05794-0.

## Introduction

According to the 2017 World Workshop classification, stages III or IV, grade C periodontitis, formerly defined as aggressive periodontitis, is characterized by the rapid progression of a particularly severe form of periodontal disease [1]. Its advanced and rapidly progressing tissue destruction pattern and the poorly elucidated mechanism of host response make the clinical management of this form more complex [2]. The current EFP S3-Level Clinical Practice Guideline recommended the use of adjunctive measures such as systemic antibiotics for this specific patient category [3, 4]. Indeed, taking into account the public health concern related to the increase in antibiotic resistance and the efficacy of site-specific infection/inflammation management strategies, local application of antimicrobials, photo/mechanical and physical means can provide benefits to conventional periodontal treatment [2, 3, 5, 6].

Favorable outcomes of adjunctive interventions to non-surgical mechanical instrumentation in young individuals with grade C periodontitis have been indicated in several studies [7–9]. However, subgingival instrumentation, with or without adjunctive therapies, has been suggested to be insufficient in patients with deep periodontal pockets [3]. Therefore, surgical periodontal therapies incorporated with regenerative procedures may be required to eliminate deep residual pockets, reconstruct the intrabony part of the defects, and prevent tooth loss [3]. Regenerative surgical procedures have been shown to be efficacious for the treatment of intrabony defects in patients with severe periodontitis, with the rapid rate of progression [10].

Within the visible red or near-infrared (NIR) range of the spectrum (600 to 700 nm and 780 to 1100 nm), low-level lasers (LLL) or light-emitting-diodes (LEDs) have been widely utilized as an adjunct therapy for periodontitis treatment based on their photobiomodulation and decontamination effects, which primarily occur at the level of cellular respiratory chain [11, 12]. These modalities promote mitochondrial activity and activate the mechanisms, i.e., inducing intracellular metabolic changes, enhancing the cellular resuscitation system, and increasing adenosine triphosphate (ATP) and extracellular matrix (ECM) production [11, 13]. LLLs or LEDs in conjunction with photosensitizing agents (optical absorption dye) have been referred to as antimicrobial photodynamic therapy (aPDT). This procedure stimulates the dye to form free radicals of singlet oxygen that will act as toxic to the target cells or bacteria mainly as a result of deterioration to the cytoplasmic membrane and DNA, [14] thereby demonstrating anti-microbial activity at periodontal pathogenic bacteria in combination with periodontal treatment [15]. It has also been noted to have an impact on vascularization and new bone formation by modulating or activating cell metabolism in the surrounding tissues through photobiomodulation [16, 17]. Similar to the biostimulatory features of LLLs or LEDs, gaseous ozone therapy applications have been recently exploited because of their analgesic, immunomodulatory, and anti-inflammatory effects, which may have the ability to provide significant added benefits in periodontal healing [18, 19]. However, in the literature, limited information exists regarding the efficacy of these modalities adjunct to the periodontal regenerative therapies on the processes and sequences of the healing and, consequently, in the postoperative expression levels of inflammation, angiogenesis, and osteogenesis biomarkers. Therefore, the objectives of the present study was: (i) to investigate the additional influence of multiple sessions of aPDT, LED photobiomodulation, and topical gaseous ozone therapy applications associated with surgical regenerative treatments by using an allogenic bone graft in combination with a collagen membrane on clinical and patient-centered outcomes in patients with stage III/IV, grade C periodontitis, and (ii) to analyze mRNA expression levels of vascular endothelial growth factor (VEGF), interleukin − 6 (IL-6), runt-related transcription factor 2 (RunX2), NEL-like 1 (Nell-1), and osterix in gingival crevicular (GCF) samples at baseline and the 1-, 3- and 6-month follow-ups after the treatment procedures. The null hypothesis of the study was that aPDT, LED photobiomodulation, and topical gaseous ozone would provide the same clinical attachment level (CAL) values as the regenerative treatment without any local adjunctive applications (control group).

## Materials and methods

### Study design and patient selection

The present study was a single-blinded, parallel-group, superiority randomized controlled clinical trial (ClinicalTrials.gov identifier: NCT05447026) with a 6-month follow-up and compliant with the principles of the Helsinki Declaration of 1975, as revised in 2013. The protocol was approved by the Clinical Investigation Ethics Committee of Ankara University’s Faculty of Dentistry (ID 36290600/105) and also the Republic of Turkey Ministry of Health, Turkey Pharmaceuticals and Medical Devices Agency (No: 71146310-511.06-E.199,784 Subject: 2017–064).

Patients diagnosed with localized/generalized periodontitis stage III or IV, grade C at the Department of Periodontology, Faculty of Dentistry, Gazi University, between January 2019 and May 2021, were considered eligible for this study. A detailed information sheet about the study was given to all participants, and written informed consent was obtained before their participation.

The patients exhibited the following clinical and radiographic features: the presence of at least two non-adjacent interproximal sites with interdental CAL ≥ 5 mm, probing pocket depths (PD) ≥ 6 mm, radiographic bone loss extending to mid-third of root or beyond, and bleeding on probing (BOP) ≥ 30%. Patients were considered the generalized type of periodontitis in case of the presence of 30% or more of the teeth had CAL ≥ 5 mm [1]. Regarding the periodontitis grade, patients with bone loss/age coefficient > 1.0 were assigned as grade C [1].

The following inclusion criteria were used: 1) ≤ 35 years of age; 2) presence of ≥ 12 teeth distributed in all four quadrants; 3) presence of residual periodontal pockets [i.e., probing depth (PD) ≥ 6 mm] 2 months after the completion of step 1 and 2 periodontal treatment 4) presence of inter-proximal defects on a single-rooted tooth or molars in either the maxilla or the mandible without extension to the furcation area and associated to an intrabony defects of ≥ 3 mm as detected in periapical radiographs (contained or non-contained defects); 5) full-mouth plaque score (FMPS) and full-mouth bleeding score (FMBS) ≤ 20%; 6) absence of degree 2 or 3 mobility.

The exclusion criteria were as follows: (1) females that were pregnant or breastfeeding; (2) systemic disease that could affect the risk or progression of periodontal disease, such as uncontrolled diabetes mellitus (haemoglobin A1c ≥ 7.0%); (3) medications that significantly impact periodontal inflammation and bone metabolism (i.e., bisphosphonates, selective serotonin reuptake inhibitors [SSRIs], proton pump inhibitors [PPIs], calcium channel blockers (CCBs), benzodiazepines, and corticosteroids); (4) smokers or use of other tobacco products; (5) systemic antimicrobial treatment (up to 3 months prior study inclusion); and (6) undergoing periodontal surgery at the experimental sites.

### Study groups, randomization and allocation

All patients received steps 1 and 2 of periodontal therapy according to the S3 level guideline, [3] including proper oral hygiene practices, risk factor control, and supra- and subgingival instrumentation. Three months after the completion of these treatments, the response to second step of therapy was assessed, and surgical procedures with adjunctive therapies were performed in the presence of residual pockets (PD ≥ 6 mm with BOP). Patients were randomly assigned to one of the adjunctive treatment modalities utilizing a computer-generated random block design by the biostatistician of the study (O.K).

According to the treatment strategies, the patients were randomly divided into four groups: (i) Control group, (ii) aPDT group, (iii) Photobiomodulation group, and (iv) Ozone group.

Each patient was assigned a study code by a researcher (AU), who was masked for the treatment procedures, clinical measurements, and sample collections. Opaque-sealed envelopes with the allocation groups were opened at the end of surgery by the surgeon, and adjunctive treatments were performed.

### Surgical approaches

The surgical procedures were performed by the same experienced researcher (S.C.I.), and were identical for all the study groups. Following local anesthesia, the experimental sites were treated with a simplified papilla preservation technique [20] to access all tooth surfaces for adjunctive measures. Granulation tissue was removed, and direct instrumentation of the affected root surfaces was performed under saline irrigation. Intrabony defects were filled with granules of allograft bone material (Maxgraft^®^ cancellous granules, Botiss Biomaterials GmbH, Berlin, Germany, part of Straumann Group, Basel, Switzerland) and covered with a native porcine pericardial collagen membrane (Jason^®^, Botiss Biomaterials). The flaps were repositioned and sutured without any tension in order to achieve primary closure of the inter-dental area using a 5 − 0 mono-filament non-resorbable PTFE suturing material (Profimed, Medipac^®^ Kilkis, Greece).

The following measurements were recorded at the time of surgery upon completion of intrasurgical debridement: (1) the thickness of the primary flap, evaluated at 1.5 mm apical to the coronal border of the flap with a caliper (Alpha Tools Digital Caliper, Mannheim, Germany) accurate to the nearest 0.1 mm; [21] (2) intrabony defect type (2- or 3-walled defects or combinations); (3) defect depth, the distance from the bone crest to the bottom of the bone defect; (4) defect width, the distance from the most coronal point of the bony walls surrounding the defect to the root surface; (5) the distance between CEJ and the bottom of the defect; and (6) the distance between CEJ and the coronal part of the defect.

### Postoperative instructions

All included patients received the same written postoperative treatment instructions, and the postoperative care consisted of using mouth rinse containing 0.12% chlorhexidine gluconate and benzydamine hydrochloride (Kloroben, Drogsan, Istanbul, Turkey) twice a day and instructing to discontinue toothbrushing and interdental cleaning around the surgical sites for two weeks. Flurbiprofen 100 mg (Majezik, Sanovel Pharmaceuticals, Istanbul, Turkey), twice daily, as needed to control postoperative pain, and Amoxicillin 500 mg (Largopen 500 mg tablet, Bilim Ilaçlari A.S, Istanbul, Turkey), three times daily, were prescribed for five days to minimize the risk of postoperative complications. Sutures were removed two weeks after surgery. Recall appointments were scheduled once a week during the first month postoperatively and every 3 months thereafter.

### Adjunctive therapies protocol

All the adjunctive therapies were performed by the surgeon (S.C.I.). aPDT group received an additional application of a diode laser with a wavelength of 970 ± 15 nm and a power rating of 2 W (continuous mode) (SiroLaser Xtend; Sirona Dental Systems GmbH, Bensheim, Germany) at the time of surgery and on the 1st, 3rd, and 7th postoperative days [22]. Indocyanine-green (ICG) as a photosensitizer (Periogreen^®^, Elexxion AG, Singen, Germany) at a concentration of 1 mg/ml was applied all around the tooth for 3 min and surgical site on the buccal and the lingual sides of the flaps. After suture application, irradiation was performed with a 400 μm diameter optical fiber probe in non-contact mode for 30 s for each of four sites of the tooth and two points-buccal and lingual/palatal interdental papilla sites, slowly moving the light spot on the target area, with the energy density of 8.6 J/cm^2^. All the sites were thoroughly rinsed with sterile saline to remove any excess photosensitizer liquid.

In the LED photobiomodulation group, irradiation was carried out with a LED device (OsseopulseTM AR 300, Biolux Research, Vancouver, British Columbia, Canada) with a wavelength of 626 nm in the near-infrared region at a dose of 20 mw/cm^2^ at the time of surgery, and on the 1st, 3rd, and 7th postoperative days for 20 min with a total energy of 222 J and energy density of 46.2 J/cm^2^ [23] After suture application, the LED device was positioned on the buccal aspect of the surgical area, and the irradiation was applied transcutaneously.

Ozone group received topical ozone application with an ozone generator (OzoneDTA generator, APOZA, Taiwan) at 80% concentration using probe #3 for 30 s per site, [24] defined as the same sites of aPDT group at the time of surgery and on the 1st, 3rd, and 7th postoperative days. The sites in the control group received only saline irrigation for 2 min in 4 sessions with the same intervals as the other study groups.

### Clinical examination

The following periodontal clinical parameters were measured at six sites of each tooth (mesiobuccal, midbuccal, distobuccal, mesiolingual/palatal, midlingual/palatal, and distolingual/palatal) with a manual periodontal probe (UNC 15 probe Hu- Friedy, Chicago, IL, USA): plaque index (PI), [25] gingival index (GI), [26] BOP, [27] PD, CAL, and gingival recession (GR). The measurements were rounded to the nearest 0.5 mm. At baseline (T0), and 3 (T3) and 6 months (T6) follow-up periods, all the measurements were performed by the same calibrated examiner (J.S.), who was blinded for the patient group assignment. Calibration was performed by measuring PD and CAL in ten patients with two contralateral teeth having PD and CAL ≥ 5 mm on proximal sites. Each patient was examined twice at a 48-hour interval and calibration was accepted if the similarity of the two measurements reached a level of > 90% (Cohen’s Kappa analyses: mean intra-examiner reliability PD: 0.91, CAL: 0.89).

Early wound healing index (EHI) was evaluated at the level of the interdental papilla according to Wachtel et al. [28] at 2 weeks after surgery. The number of membrane exposures and buccolingual extension of the exposure (mm) were recorded during postoperative two weeks. Postoperative swelling was assessed using four categorical scores (i.e., 0: absent, 1: slight, 2:moderate, 3: severe), [29] and patients’ postoperative morbidity regarding pain and discomfort was evaluated on a visual analog scale (VAS) ranging from 0 (no) to 10 (very severe) at the 1st, 3rd, 5th, 7th, and 15th postoperative days.

### Gingival crevicular fluid sample collection and mRNA expression of VEGF, IL-6, RunX2, Nell-1, and osterix

GCF sampling was performed by two filter paper strips (Periopaper; Oraflow Inc., New York, NY, USA), which were gently inserted 1 to 2 mm into the pocket in the interdental defect sites (mesial or distal) and left there for 30 s. The sampling was done at T0, 1 month (T1), 3 months (T3), and 6 months (T6) follow-up periods. The volumes of the samples were measured with a calibrated device (Periotron 8000, Proflow Inc., Amityville, NY, USA). The strips were placed in one propylene tube and were frozen immediately at -80 °C until RNA extraction. Total RNA isolation from GCF samples was performed by using purification kit (RiboEx (301-001) and Hybrid-R (305 − 101) Geneall Biotechnology Co., Seoul, Korea) according to the manufacturer’s recommendations. Isolated RNA samples were reversely transcribed by using random primers with reverse transcriptase (WizScript™ III Reverse Transcriptase, Geneall Biotechnology Co., Seoul, Korea). Reverse transcription reactions were performed according to the following conditions: 10 min at 25 °C, 120 min at 37 °C, 5 min at 85 °C, and 4 °C hold.

The mRNA levels of VEGF, IL-6, RunX2, Nell-1, and osterix were determined using a real-time polymerase chain reaction (rt-PCR) system (Applied Biosystems™ 7500 Fast Real-Time PCR, Foster City, CA, USA) and a specific kit (WizPure™ qPCR Master (SYBR), Geneall Biotechnology Co.). The target gene and the probe sequence of each specific TaqMan Gene Expression Assay primer sequences were presented in Supplemental T1).

The quantification of relative amounts of mRNA expressions were performed with ACTB as the internal reference gene using “∆∆Ct Method”. ΔΔCt values ​​were calculated as 2^−ΔΔCt^ as relative expression (fold change). Relative expression values of each gene are expressed relative to the GCF sample with the lowest expression, which was set to 1. If the fold change value was above 1, it was interpreted that the mRNA expression of the target group increased relative to the mRNA expression of the control group. If not, the mRNA expression of the target group was considered as reduced compared to the mRNA expression of the control group.

### Outcomes measures

The primary outcome variable of the study was defined as changes in CAL at T6. Secondary outcome measures were BOP, PD, GR, EHI, VAS pain and discomfort, and mRNA levels of VEGF, IL-6, RunX2, Nell-1, and osterix.

### Statistical methods

A sample size of each group of 10 subjects was calculated to attain a power of 80% with an effect size of 0.25, using a Repeated Measures ANOVA (four groups, alpha = 0.05, non-sphericity correction) with a 0.05 significance level to detect a difference of 0.56 mm in CAL between the aPDT and control groups [30].

One-way ANOVA or Kruskall Wallis H test was used for independent group comparisons, depending on the distributional properties of the data. If significant differences were detected between the groups, the Bonferroni post-hoc test was utilized. Chi-square test was used for proportions, and its counterpart, Fisher’s Exact test, was used when the data were sparse. The difference between the four groups, time points, and the interaction of these two main effects were tested with two-way repeated measures of ANOVA. The sphericity assumption was performed by using Mauchly’s test sphericity. As the violation of this assumption, Wilk’s Lambda statistic was used as multivariate test results. When the p-value from the ANOVA test statistics was statistically significant, pairwise comparisons were used to know which time point differed from which others, and the results were summarized using estimated means, standard errors and 95% CI’s. Correlative relationships between the cytokines and the changes in clinical parameters were assessed by the Pearson correlation coefficient. To assess the effect of treatment methods on the primary outcome with possible indicators (i.e., initial PD > 7 mm, thickness of the primary flap, intrabony defect type and intrabony defect depth and width) generalized linear model (GLM) was performed. A p-value of < 0.05 was considered statistically significant. All statistical analyses were done using SPSS (IBM Corp. Released 2015. IBM SPSS Statistics for Windows, Version 23.0. Armonk, NY: IBM Corp.).

## Results

**Fig. 1:**
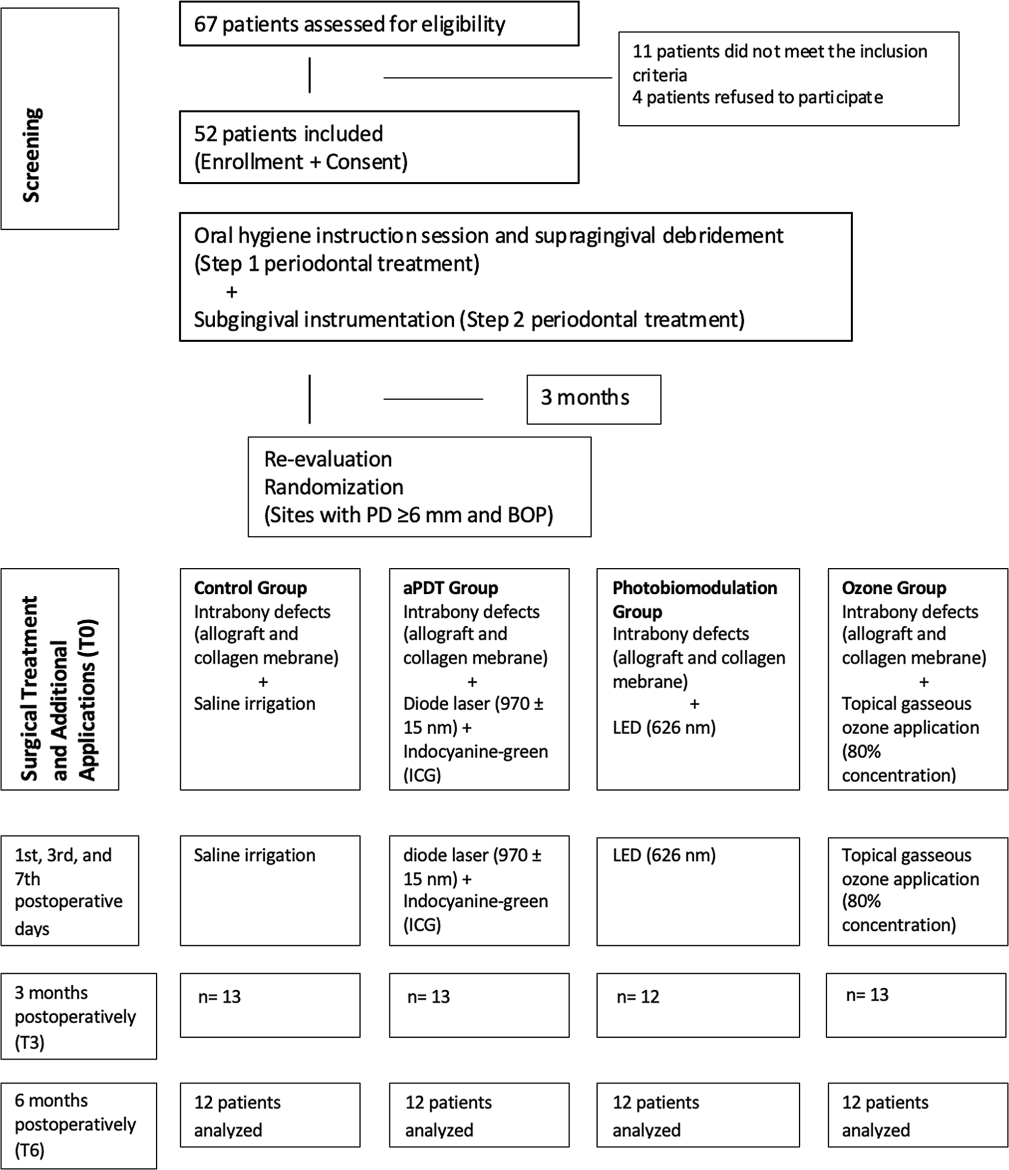
Flowchart of the study design

Sixty-seven patients were initially selected for the study, of which 11 did not meet the inclusion criteria, and four refused to participate. Eventually, systemically healthy and non-smoker 52 patients (32 females and 20 males) were included in the study. Of the initial 52 patients, 4 patients could not attend the follow-up periods properly and were excluded for the final analysis. A total of 48 patients (29 females and 19 males) with a mean age of 31.45 ± 5.92 years could be followed over the study period. The study flowchart was presented in Fig. 1. The characteristics of the study population and defect area were summarized in Table 1. No severe complications or adverse events were observed after the surgical procedures and adjunctive therapies. During the postoperative two weeks, membrane exposure was observed in three patients (25%) for the control group, two patients in the photobiomodulation group (16.6%), and five patients in the ozone group (41.6%). Whereas, none of the patients in aPDT group showed membrane exposure. At postoperative two weeks, five defects showed EHI = 1, three defects showed EHI = 2 and 2 defects showed EHI = 3 in the control group, while in ozone group, five defects showed EHI = 1, and five defects were EHI = 2. In the photobiomodulation group, eight defects exhibited EHI = 1, and all the defects were EHI = 1 for aPDT group. A significant relationship was found between EHI and treatment methods (*p* = 0.006), demonstrating a significant difference between control and aPDT groups.

**Table 1:** The characteristics of the study population and defect area

Patient Characteristics	Control Group	aPDT Group	Photobiomodulation Group	Ozone Group	*p* value
Age (years)	31.3 ± 6.17	31.6 ± 7.15	30.6 ± 6.15	31.3 ± 5.97	0.995^a^
Female Gender (n/%)	9/31.0%	6/20.7%	5/17.3%	9/31.0%	0.323^b^
Diagnosis (n/%) Stage III grade C Stage IV grade C	8/26.6% 4/20.0%	6/23.4% 6/30.0%	6/23.4% 6/30.0%	8/26.6% 4/20.0%	0.714^b^
**Defect Area Characteristics**					
Tooth (n/%) Incisors Premolars Molars	0/0.0% 6/35.3% 6/23.1%	0/0.0% 5/29.4% 7/26.9%	2/40% 3/17.7% 7/26.9%	3/60.0% 3/17.7% 6/23.1%	0.319^b^
Initial PD category (n/%) Moderately deep pockets (PD≥ 6 mm) Deep pockets (PD>7 mm)	4/20.0% 8/28.6%	6/30.0% 6/21.4%	5/25.0% 7/25.0%	5/25.0% 7/25.0%	0.969^b^
The thickness of the primary flap (mm)	2.28 ± 0.81	2.49 ± 0.40	2.54 ± 0.90	2.35 ± 0.49	0.801^a^
Intrabony defect type (n/%) 2-wall defect 3-wall defect	8/23.5% 4/28.6%	8/23.5% 4/28.6%	9/26.5% 3/21.4%	9/26.5% 3/21.4%	0.999^b^
Intrabony defect depth (mm)	3.60 ± 0.70	3.80 ± 0.92	4.0 ± 0.67	4.0 ± 0.91	0.611^a^
Intrabony defect width (mm)	3.25 ± 1.18	3.40 ± 0.70	3.70 ± 1.06	2.80 ± 0.59	0.167^a^
The distance between CEJ and the bottom of the defect (CEJ-BD) (mm)	5.50 ± 0.85	5.65 ± 0.85	5.95 ± 1.14	5.85 ± 1.73	0.812^a^
The distance between CEJ and the coronal part of the defect	2.10 ± 0.94	2.05 ± 0.57	2.35 ± 0.58	2.15 ± 0.67	0.742^a^

*p<0.05 considered statistically significant

a; Kruskal Wallis H test, b; Fisher’s exact test

PD; Probing pocket depths, CEJ: Cemento-enamel junction

### Clinical parameters

All the periodontal clinical parameters registered at T0, T3, and T6 were presented in Table 2. The only significant difference among the treatment groups was identified for GR, which was between control and photobiomodulation groups (*p* = 0.041). When analyzing time*group interactions, PI and PD showed statistically significant differences (*p* = 0.034 *p* = 0.022, respectively). PI showed a decreasing trend at T3 compared to baseline in all the groups, but increased at T6 compared to T0 and T3, except for photobiomodulation group. In control group, PI indicated a statistically significant increase at T6 compared with the T3 value (*p* = 0.007). All the groups were homogeneous at T0 regarding PD and CAL, demonstrating a greater reduction between T0 and T3 and between T0 and T6. When the changes of the groups according to time regarding PD were examined, statistically significant differences were noted between T0 and T3 and between T0 and T6 for all the groups (*p* < 0.001; *p* < 0.001, respectively). Similar to PI values, control group was the only group that revealed a statistically significant increase for PD at T6 compared to the T3 value (*p* < 0.001). However, according to the time*group interaction, CAL could not be identified as significant (*p* = 0.487). The mean CAL gain at T6 was 1.48 ± 0.59 mm for the control group, 1.80 ± 0.85 mm for aPDT group, 2.08 ± 0.56 mm for photobiomodulation group, and 1.68 ± 0.92 mm for ozone group. Regarding the comparison of PD and CAL among the groups according to initial PD category [moderately deep (≥ 6 mm) and deep (> 7 mm)], no significant difference was observed for the sites with initial moderately deep PD. Nevertheless, significant differences were noted for initial deep PD sites between control and photobiomodulation groups for PD at T6 (*p* = 0.011). In terms of CAL, significant differences were identified between control and aPDT groups at T3 (*p* = 0.037), and between control and aPDT, and control and photobiomodulation groups at T6 (*p* = 0.007; *p* = 0.022, respectively) (Supplemental T2).

**Table 2:** Comparison of the periodontal clinical parameters among the groups

Clinical Parameters	Control Group	aPDT Group	Photobiomodulation Group	Ozone Group	Group effect p^*^ value	Time effect *p*^‡^ value		
						T0-T3	T0-T6	T3-T6
PI Baseline (T0) 3 months (T3) 6 months (T6)	0.30 ± 0.12 0.23 ± 0.06 0.45 ± 0.08	0.18 ± 0.12 0.18 ± 0.06 0.25 ± 0.08	0.33 ± 0.12 0.25 ± 0.06 0.20 ± 0.08	0.43 ± 0.12 0.15 ± 0.06 0.30 ± 0.08	0.570	0.139	0.999	**0.018**
**Group by time interactions** ***p*** ^***†***^ **value** ** T0-T3** ** T0-T6** ** T3-T6**	0.999 0.890 **0.007**	0.999 0.999 0.842	0.999 0.999 0.999	**0.034** 0.999 0.105				
GI Baseline (T0) 3 months (T3) 6 months (T6)	0.05 ± 0.04 0.18 ± 0.06 0 ± 0	0.05 ± 0.04 0.07 ± 0.03 0 ± 0	0.02 ± 0.02 0.15 ± 0.05 0.05 ± 0.03	0.02 ± 0.02 0.12 ± 0.05 0.02 ± 0.02	0.451	0.075	0.541	**<0.001**
**Group by time interactions** ***p*** ^***†***^ **value**	0.194							
BOP Baseline (T0) 3 months (T3) 6 months (T6)	70.0 ± 5.85 15.0 ± 3.75 10.0 ± 5.22	55.0 ± 5.85 5.0 ± 3.75 5.0 ± 5.22	65.0 ± 5.85 20.0 ± 3.75 20.0 ± 5.22	67.5 ± 5.85 12.5 ± 3.75 17.5 ± 5.22	0.072	**<0.001**	**<0.001**	0.999
**Group by time interactions** ***p*** ^***†***^ **value**	0.427							
PD Baseline (T0) 3 months (T3) 6 months (T6)	4.35 ± 0.24 2.58 ± 0.13 2.80 ± 0.15	3.98 ± 0.24 2.23 ± 0.13 2.28 ± 0.15	4.23 ± 0.24 2.15 ± 0.13 2.15 ± 0.15	4.13 ± 0.24 2.18 ± 0.13 2.25 ± 0.15	0.103	**<0.001**	**<0.001**	**0.002**
**Group by time interactions** ***p*** ^***†***^ **value** ** T0-T3** ** T0-T6** ** T3-T6**	**<0.001** **<0.001** **<0.001**	**<0.001** **<0.001** 0.876	**<0.001** **<0.001** 0.999	**<0.001** **<0.001** 0.353				
GR Baseline (T0) 3 months (T3) 6 months (T6)	0.20 ± 0.04 0.20 ± 0.05 0.28 ± 0.05	0.10 ± 0.04 0.15 ± 0.05 0.15 ± 0.05	0.03 ± 0.04 0.05 ± 0.05 0.05 ± 0.05	0.03 ± 0.04 0.05 ± 0.05 0.10 ± 0.05	**0.041**	0.141	**0.007**	0.056
**Group by time interactions** ***p*** ^***†***^ **value**	0.267							
CAL Baseline (T0) 3 months (T3) 6 months (T6)	4.55 ± 0.25 2.78 ± 0.17 3.08 ± 0.18	4.08 ± 0.25 2.35 ± 0.17 2.40 ± 0.18	4.28 ± 0.25 2.20 ± 0.17 2.20 ± 0.18	4.15 ± 0.25 2.33 ± 0.17 2.35 ± 0.18	0.068	**<0.001**	**<0.001**	0.060
**Group by time interactions** ***p*** ^***†***^ **value**	0.487							

The data are presented as the mean±standard error of the mean. *p*<0.05 considered statistically significant and shown in bold. The significance of group and time effects, and time*group interactions were assessed by repeated-measures ANOVA. *, intergroup comparisons; ^†^ time*group interactions; ^‡^, the changes of the groups according to time. In case of *p*<0.05, pairwise comparisons were performed by using Bonferroni post-hoc test

PI; Plaque index, GI; Gingival index, BOP; Bleeding on probing, PD; Probing pocket depths, GR; Gingival recession, CAL; Clinical attachment level

The effect of treatment methods incorporating the possible confounding variables on CAL gain was assessed by GLM model (Table 3). According to this model, the initial PD category revealed a statistically significant relationship with the primary outcome. PD > 7 mm presented a statistically significantly higher CAL gain (estimated coefficient 0.56) than those with PD ≥ 6 mm sites (*p* < 0.001). An estimated generalized linear model can be shown by $y=-\text{0,87}+\left(\text{0,56}\right){x}_{i}$.

**Table 3:** The effect of treatment methods incorporating the possible confounding variables on the primary outcome

Parameter		Std. Error	95% Wald Confidence Interval		
	B		Lower	Upper	*p*
(Intercept)	-0.87	0.50	-1.85	0.12	0.08
The initial PD>7 mm	0.56	0.12	0.32	0.80	**0.00**
Treatment group (aPDT)	0.24	0.14	-0.04	0.53	0.09
Treatment group (Photobiomodulation)	0.20	0.14	-0.08	0.48	0.16
Treatment group (Ozone)	-0.01	0.16	-0.32	0.29	0.94
The thickness of the primary flap	-0.09	0.09	-0.26	0.08	0.30
Intrabony defect depth	0.12	0.08	-0.04	0.28	0.13
Intrabony defect width	0.07	0.06	-0.04	0.18	0.22
Intrabony defect type	-0.03	0.12	-0.28	0.21	0.79

Control group was the reference for treatment group

### mRNA expression levels of biomarkers

**Fig. 2:**
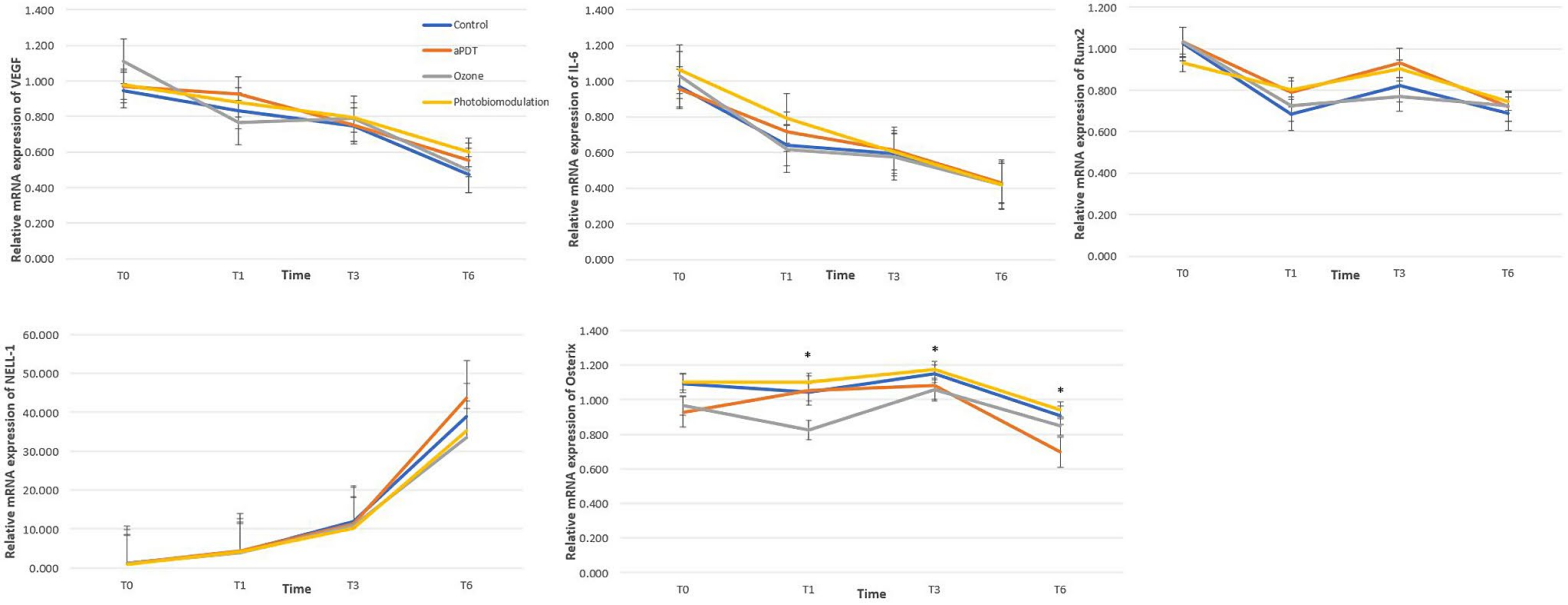
The relative mRNA expression levels of VEGF, IL-6, RunX2, Nell-1, and osterix over time for study groups. The intergroup comparisons among the groups were were assessed by repeated-measures ANOVA. ^*^p < 0.05 considered statistically significant. In case of *p* < 0.05, pairwise comparisons were performed by using Bonferroni post-hoc test

Figure 2 depicted the groups’ relative mRNA expression levels of VEGF, IL-6, RunX2, Nell-1, and osterix over time. The mRNA levels of IL-6 were significantly reduced at each time point within all the groups (*p* < 0.001). No significant changes were detected regarding intergroup comparisons at any follow-up periods. The mRNA levels of VEGF exhibited statistically significant reductions at each time point within the groups in control and aPDT groups (*p* < 0.001). Photobiomodulation group showed a significant decrease for VEGF levels only between T0 and T6 and between T1 and T6 (*p* < 0.001; *p* = 0.009, respectively). In ozone group, a slight increase was noted between T1 and T3, unlike the changes between other time points for all the groups. Although not significant, the lowest level of VEGF expression was observed in control group at T6 among all the groups. The relative osterix mRNA levels showed statistically significant difference among the treatment groups (*p* = 0.014). The differences between the control and aPDT, control and ozone, photobiomodulation and aPDT, and photobiomodulation and ozone groups were identified as significant (*p* = 0.004, *p* = 0.012, *p* = 0.012; *p* = 0.001, respectively). For all the groups, an increasing trend was noted for the osterix expression at T3, which demonstrated the greatest expression levels according to all study follow-ups, and then the levels decreased back to baseline levels at T6. Only aPDT group showed an expression tended to increase at T1 compared to that at T0, while other groups exhibited decreased osterix levels at T1 compared to baseline. Regarding the change over time, only control group did not show a statistically significant upregulation at T3 compared to other follow-ups. The relative mRNA levels of RunX2 and Nell-1 did not differ significantly for intergroup comparisons or time*group interactions (*p* > 0.05). All the groups had greater increments in the mRNA level of Nell-1 at T6 compared to the other follow-up periods. Although not significant, the highest level of Nell-1 expression was observed in aPDT group at T6 among all the groups. On the contrary, the mRNA level of RunX2 exhibited a decrease at T6 despite presenting a slight increase at T3 according to the T0 values.

Correlations of the relative mRNA levels of biomarkers at T6 and the changes in clinical parameters between T0 and T6 were presented in Table 4. There were significantly moderate positive correlations between the mRNA expression levels of VEGF and osterix and between PI changes from T0 to T6 and IL-6 levels and RunX2 levels (*p* < 0.05). A moderate positive correlation was also identified between osterix level, CAL gain, and PD reduction at T6 (*p* < 0.05).

**Table 4:**
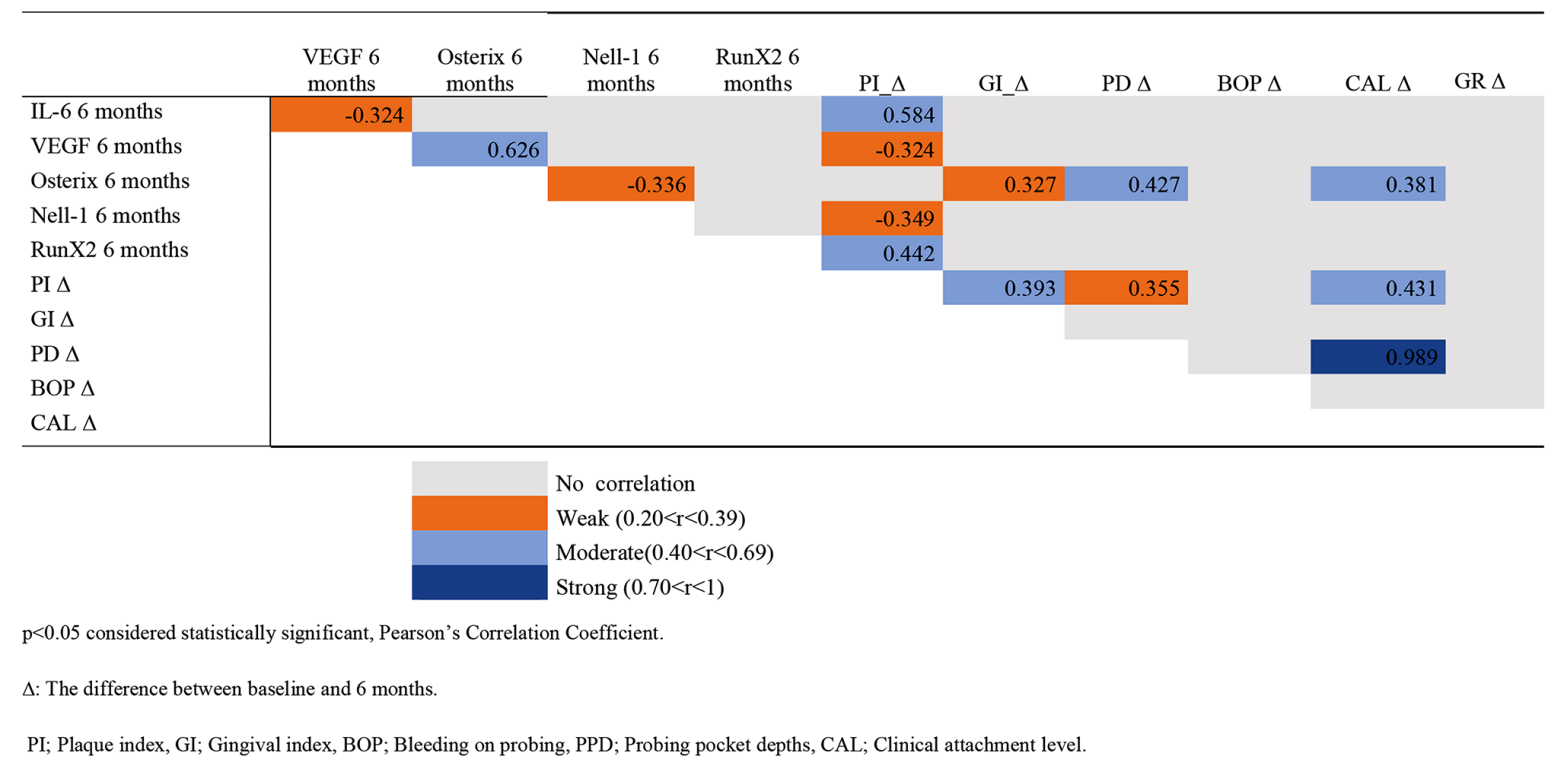
Correlations of the relative mRNA levels of biomarkers and the changes in clinical parameters at 6 months postoperatively

### Patient-reported outcome measures (PROMs)

No significant difference was revealed among the groups regarding postoperative swelling, pain, and discomfort. On the 1st day postoperatively, slight swelling was seen in seven patients (58.3%) in the control group and five in the ozone group (41.6%). At one week, only the ozone group exhibited slight swelling in two patients (16.6%), while swelling was not observed in any patient in the other groups. On the 1st day postoperatively, the highest VAS mean scores of pain and discomfort were noted for the control group (4.70 ± 1.05; 3.40 ± 0.85, respectively). On the 5th day, no pain was reported for aPDT and photobiomodulation groups, while postoperative pain was recorded for ozone and control groups.

## Discussion

The results of the present study indicated that all the treatment modalities associated with surgical regenerative treatments in patients with stage III/IV grade C periodontitis resulted in significant clinical improvements at 6 months postoperatively compared to the baseline. No statistically significant difference could be demonstrated for clinical parameters except GR among aPDT, LED photobiomodulation, and topical gaseous ozone therapy. Therefore, considering the primary outcome (i.e., CAL), the null hypothesis of the study was accepted. A statistically significant difference in GR was revealed in favor of the LED photobiomodulation group compared to the control group. Besides, for the sites with initial deep PD, additional use of aPDT and LED photobiomodulation presented a significantly beneficial effect on CAL and PD. Regarding the results of quantitative analysis of cytokines mRNA expression, the osterix levels indicating a significant difference among treatment methods in favor of the photobiomodulation group seemed to be related to CAL gain after regenerative treatment of intrabony defects in patients with stage III/IV grade C periodontitis.

In the management of residual pockets associated with intrabony defects in stage either III or IV and grade C periodontitis, the use of biomaterials for regenerative therapy was indicated to provide better outcomes than open flap debridement (OFD) alone, despite being associated with a relatively high degree of variability [10, 31]. A recent systematic review and meta-analysis by Diaz-Faes et al. [10] reported a mean CAL gain of 0.66 mm and a reduction in PD of 1.00 mm with regenerative surgical therapy for intrabony defects in stage III or IV grade C periodontitis patients at 6 months. Compared with these findings, the present study indicated slightly better results in terms of CAL gain (1.48 ± 0.59 mm) and PD reduction (1.55 ± 0.58 mm) after the regenerative surgical treatment without any additional applications (control group). These differences could be related to presurgical clinical and inflammatory conditions and postsurgical rates of interproximal wound dehiscence with membrane exposure, which has been related to significantly reduced clinical improvements regarding CAL gain and PD reduction [32]. Indeed, the early postoperative healing phase, i.e., blood clot formation and cell migration and proliferation, play an essential role in achieving successful outcomes in surgical periodontal procedures [12]. Utilizing adjunctive modalities, such as lasers, has been shown favorable outcomes in various surgical periodontal approaches in efforts to improve early postoperative wound healing and decrease patients’ postoperative morbidity [11]. In parallel with this, the control group of this study exhibited higher rates of membrane exposure and lower EHI scores and presented less favorable clinical outcomes and PROMs compared to the aPDT and LED photobiomodulation groups. However, ozone group showed the highest rates of membrane exposure among the groups.

Photobiomodulation and aPDT mediated by LLL or LED have been mostly explored in periodontitis treatment and demonstrated significant clinical improvements, elimination of periodontal pathogens, and reduced inflammatory mediators [2, 30, 33–35]. On the other hand, some studies have failed to achieve the adjunctive benefit of these therapeutic resources [15, 36]. It has been established by the American Academy of Periodontology best-evidence consensus review that the evidence to support the use of aPDT as an adjunctive treatment could not present a clinically relevance improvement in PD and CAL compared to conventional periodontal therapy alone in patients with moderate/severe periodontitis [37]. Partially compatible with this finding, the present study failed to show any significant difference in clinical parameters except for GR among different adjunctive applications in conjunction with GTR in comparison to GTR procedures alone, although reflecting an average trend in favor of adjuvant therapies. Nonetheless, particularly for sites with baseline PD > 7 mm, aPDT and photobiomodulation groups exhibited significantly better PD and CAL results than the control group. A possible explanation for the more pronounced effect of adjunctive applications in deeper periodontal defects could lead to deeper microbial reduction and improved periodontal wound healing response. Furthermore, high strength of evidence has shown that the regenerative treatments of deeper, narrower defects and defects with more walls positively influence CAL gain and radiographic bone defect filling [38]. Regarding GR, which was observed to be only statistically significant in the comparison between groups, LED photobiomodulation group exhibited more favorable outcomes compared to the control group. This finding could be related to the stimulatory effect of photobiomodulation at the cellular level by stimulating the development of new capillaries and enhancing blood flow in the injured area, which leads to faster tissue healing through improved oxygen intake [18, 39].

At a cellular and molecular level, photobiomodulation and aPDT approaches have been shown to stimulate the proliferation and osteoblastic differentiation of periodontal ligament stem cells (PDLSCs) [39–41]. Moreover, these adjunct modalities have been suggested to promote bone healing and regeneration in the defects grafted with osteoconductive/osteoinductive biomaterials, particularly in the early wound healing of bone defects [42, 43]. However, the biostimulatory effect of aPDT could primarily based on through photo-activated disinfection and antimicrobial features. In a previous study, Dogan et al. [44] demonstrated that LLL therapies (LLLT) using a Nd: YAG laser with 1064 nm wavelength (100 mW, 100 mJ, 4 J/cm^2^) in combination with GTR protocols yielded favorable clinical outcomes in terms of lower GR, and greater CAL gain and PD reduction compared to GTR alone. However, an in vivo study analyzing the effect of 660 nm LED light irradiation (660 ± 25 nm, 3.5 mW/cm^2^) on the treatment of experimental periodontal intrabony defects using the combination of xenograft and barrier membrane exhibited no noticeable difference in the osteogenesis between the non-LED and LED light-irradiated specimens [12]. This result could be explained by the fact that placement of a barrier membrane can cause the attenuation of the LED light and create a limitation for activating progenitor cells from the periodontal cells and enhancing the proliferation of periodontal ligament, which is located deep inside of periodontal tissue to respond to LED irradiation.

The efficacy of repeated application or irradiation protocols compared to single application in periodontal treatment is another topic that remains contradictory [45]. A previous study by Cadore et al. [30] investigated the efficacy of multiple sessions of aPDT with a laser diode at 660 nm (maximum power of 60 mW/cm^2^ and energy density of 0.6 J/cm^2^) as an adjunct to surgical periodontal treatment and showed significant improvements in clinical outcomes compared to sham procedure. That study indicated a CAL gain of 1.97 ± 1.07 mm for aPDT at 5 months postoperatively, which was similar to the CAL gain value of the present study in aPDT group at 6 months (1.80 ± 0.85 mm) [30]. Conversely, Katsikanis et al. [15] reported no additional clinical benefit of the multiple sessions of adjunctive use of either aPDT with 670 nm GaAlAs diode laser or a diode laser with 940 nm wavelength in the non-surgical treatment of periodontitis compared to non-surgical treatment alone. It is also worthy of note that the application of LLL and LED therapies contain extreme variations to define a suitable treatment protocol (i.e., wavelength, power, amount of energy density, intervention time, number of points to be applied, frequency of treatment, and optic fibre diameter), which does not allow for an accurate comparison between the findings of the present study and previous studies.

This study investigated the effect of adjunctive applications on periodontal regenerative treatment by rt-PCR analysis of mRNA expressions of inflammatory, angiogenic, and osteogenic markers in GCF. The expression level of osterix, one of the most critical early osteogenic markers, [43] revealed a significant difference among the groups and the change over time. Photobiomodulation and control groups exhibited higher osterix expression levels than the other adjunctive modalities at all follow-up periods. Nevertheless, only aPDT group presented an increased expression trend at T1 and T3 according to T0. Osterix has been shown to be one of the downstream genes of Runx2, and both are transcriptional regulators of osteogenesis that play an essential role in promoting osteoblast differentiation and bone formation [46]. In the present study, the gene expression pattern for RunX2 had a similar trend, with osterix demonstrating up-regulated expression at T3. However, RunX2 levels failed to exhibit significant differences among the groups. On the other hand, the expressions of RunX2 tended to decrease at follow-ups compared to initial expression levels for all the groups. Nell-1 has been suggested to exhibit potent osteoinductive activity for bone regeneration by stimulating the expression of Runx2 and osterix [47]. This study demonstrated a strongly increased expression trend for the Nell-1 expressions, especially between T3 and T6, for all the groups. Although not significant, aPDT showed the highest level of Nell-1 expression, and the control group presented a greater expression level than the photobiomodulation and ozone group. This could be explained by adjunctive applications that may contribute to bone formation and maturation in the early healing period and can occur later for the control group. However, the effectiveness of aPDT on osteogenesis may have been more prolonged. The present study employed ICG of 1 mg/ml concentration and 980 nm wavelength for aPDT protocol. This wavelength has been reported to have a higher penetration depth and be effective by enhancing cell proliferation through increasing the expression of growth factors and bone repair factors such as RunX2, VEGF, and osteocalcin [48, 49].

In the literature, there is inconsistent data about the effectiveness of adjunctive use of ozone therapy on the outcomes of periodontitis treatment. Some studies exhibited statistically and clinically significant improvement of the ozone application in periodontitis treatment, [50, 51] whereas others failed to present any additional benefit compared to periodontal treatment alone [52, 53]. In the present study, ozone group did not seem superior to control group as an adjunctive application in the regenerative treatment of periodontitis, presenting no significant clinical improvement in favor of ozone group. In parallel with clinical findings, the mRNA expressions of all the selected biomarkers in ozone group did not exhibit a favorable pattern in comparison with other adjunctive modalities and control group. On the other hand, as a considerable factor related to patient demographics, female patients constituted the majority in the ozone and control groups. Although females generally have healthier behaviours in oral hygiene compared with their male peers, the significant effect of gender differences on periodontitis treatment outcome has not been clearly demonstrated in the litearture. Indeed, PI scores showed similar increase/decrease trend for all the groups during 6 months after the surgical treatments.

A limited sample size may be the main limitation of this study; however, the minimum sample size required for the study to achieve a power of at least 80% has been reached. The other limitations could be the need for microbial analyses and assessments of the potential of radiographic features. Additionally, an important limitation is that the use of systemic antibiotics prescribed to minimize the risk of postoperative complications after regenerative treatment may have obscured the effectiveness of aPDT and ozone therapy. Future studies can be conducted with different adjunctive treatment protocols to show the efficiency of these modalities. Long-term studies may also provide a better understanding of the effectiveness of these additional treatment modalities on regenerative treatment of stage III/IV grade C periodontitis.

## Conclusion

Within their limits, the present findings indicate that the additional applications of aPDT and LED photobiomodulation after regenerative treatment of intrabony defects in the patients with stage III/IV grade C periodontitis seemed to present a significant improvement in PD and CAL compared to the surgical treatment alone only for the sites with baseline PD > 7 mm. aPDT and LED irradiations also demonstrated favorable early wound healing results concerning prevention of membrane exposure and acceleration of wound closure, as well as for the patients’ perceived pain and discomfort. Osterix expressions were found to be related to CAL gain after regenerative treatment of stage III/IV grade C periodontitis independent of treatment modalities.

Control group was the reference for treatment group.

### Electronic supplementary material

Below is the link to the electronic supplementary material.

Supplementary Material 1

## Data Availability

No datasets were generated or analysed during the current study.
