# Supplementary material for: The adjunctive use of antimicrobial photodynamic therapy, light-emitting-diode photobiomodulation and ozone therapy in regenerative treatment of stage III/IV grade C periodontitis: a randomized controlled clinical trial
Source: Clin Oral Investig. 2024 Jul 12;28(8):426. doi: 10.1007/s00784-024-05794-0 (PMC11239751; doi:10.1007/s00784-024-05794-0)
Supplement: Supplementary file 1 — Supplementary Material 1 [file 784_2024_5794_MOESM1_ESM.docx]

| **Initial PPD category** | **Parameters**  **Time points** | | **Control Group** | **aPDT Group** | **Photobiomodulation Group** | **Ozone Group** | ***p* value** |
| --- | --- | --- | --- | --- | --- | --- | --- |
| **PPD≥ 6 mm** | PPD | Baseline | 3.33 ± 0.58 | 3.65 ± 0.38 | 3.75 ± 0.0 | 3.38 ± 0.43 | 0.699 |
|  |  | 3 months | 2.0 ± 0.0 | 2.20 ± 0.33 | 2.25 ± 0.29 | 2.19 ± 0.38 | 0.725 |
|  |  | 6 months | 2.08 ± 0.14 | 2.30 ± 0.45 | 2.25 ± 0.29 | 2.19 ± 0.38 | 0.867 |
|  |  | ∆ Base - 6 months | 1.25 ± 0.43 | 1.35 ± 0.55 | 1.50 ± 0.29 | 1.19 ± 0.72 | 0.451 |
|  | CAL | Baseline | 3.42 ± 0.72 | 3.80 ± 0.45 | 3.75 ± 0.0 | 3.38 ± 0.43 | 0.507 |
|  |  | 3 months | 2.08 ± 0.14 | 2.45 ± 0.57 | 2.25 ± 0.29 | 2.19 ± 0.38 | 0.766 |
|  |  | 6 months | 2.25 ± 0.43 | 2.55 ± 0.69 | 2.25 ± 0.29 | 2.19 ± 0.38 | 0.868 |
|  |  | ∆ Base - 6 months | 1.17 ± 0.29 | 1.25 ± 0.64 | 1.50 ± 0.29 | 1.19 ± 0.72 | 0.50 |
| **PPD> 7 mm** | PPD | Baseline | 4.79 ± 0.47 | 4.55 ± 0.72 | 4.54 ± 0.25 | 4.42 ± 0.92 | 0.223 |
|  |  | 3 months | 2.82 ± 0.57 | 2.10 ± 0.22 | 2.08 ± 0.13 | 2.29 ± 0.46 | 0.106 |
|  |  | 6 months | 3.11 ± 0.52 | 2.10 ± 0.22 | 2.08 ± 0.13 | 2.42 ± 0.58 | 0.007^*^ |
|  |  | ∆ Base - 6 months | 1.68 ± 0.62 | 2.45 ± 0.76 | 2.46 ± 0.29 | 2.0 ± 0.87 | 0.15 |
|  | CAL | Baseline | 5.04 ± 0.37 | 4.55 ± 0.72 | 4.63 ± 0.31 | 4.50 ± 0.99 | 0.096 |
|  |  | 3 months | 3.07 ± 0.57 | 2.05 ± 0.21 | 2.17 ± 0.13 | 2.58 ± 0.83 | 0.029^*^ |
|  |  | 6 months | 3.43 ± 0.51 | 2.05 ± 0.21 | 2.17 ± 0.13 | 2.63 ± 0.74 | 0.004^*^ |
|  |  | ∆ Base - 6 months | 1.61 ± 0.66 | 2.50 ± 0.71 | 2.46 ± 0.29 | 1.88 ± 0.88 | 0.149 |

**Supplemental Table 1.** The target gene and the probe sequence of each specific primer sequences

| **The Target Gene** | **The Probe Sequence** |
| --- | --- |
| VEGF-F | TCCACCATGCCAAGTGGTC |
| VEGF-R | GTCCACCAGGGTCTCGATTG |
| IL6-F | TACATCCTCGACGGCATCTC |
| IL6-R | ACCAGGCAAGTCTCCTCATTG |
| RUNX2-F | GTCATGGCGGGTAACGATGA |
| RUNX2-R | TGAAACTCTTGCCTCGTCCA |
| NELL1-F | CAGTGTCGGTGTCTGGAAGG |
| NELL1-R | CTGAGCCACTAAGCCGTGAA |
| Osterix-F | TCCCTGCTTGAGGAGGAAGT |
| Osterix-R | GAGTTGTTGAGTCCCGCAGA |
| ACTB-F | CATGTACGTTGCTATCCAGGC |
| ACTB-R | CTCCTTAATGTCACGCACGAT |

**Supplemental Table 2.** The comparison of PD and CAL among the groups according to initial PD category

*p<0.05 considered statistically significant, Kruskal Wallis H test.

In the sites with PPD>7 mm, the difference between the median values ​​of control-aPDT and control-ozone groups were statistically significant for PPD at 6 months (p=0.011; p=0.032).

The difference between the median values ​​of control-aPDT groups was statistically significant for CAL at 3 months (p=0.037).

The difference between the median values ​​of control-aPDT and control-photobiomodulation groups were statistically significant for CAL at 6 months (p=0.007; p=0.022).

PPD; Probing pocket depths, CAL; Clinical attachment level.
